# MADS-box family genes in sheepgrass and their involvement in abiotic stress responses

**DOI:** 10.1186/s12870-018-1259-8

**Published:** 2018-03-14

**Authors:** Junting Jia, Pincang Zhao, Liqin Cheng, Guangxiao Yuan, Weiguang Yang, Shu Liu, Shuangyan Chen, Dongmei Qi, Gongshe Liu, Xiaoxia Li

**Affiliations:** 1 Key Laboratory of Plant Resources, Institute of Botany, The Chinese Academy of Sciences, Beijing, China; 2 University of Chinese Academy of Sciences, Beijing, China; 3 College of Biological and Food Engineering, Huaihua University, Huaihua, Hunan 418000 People’s Republic of China; 4 Institute of Animal Science of Heilongjiang Province, Qiqihar, Heilongjiang China

**Keywords:** MADS-box genes, Sheepgrass, Abiotic stress, Gene expression, Sexual reproduction, Yeast two-hybrid assay

## Abstract

**Background:**

MADS-box genes are categorized into A, B, C, D and E classes and are involved in floral organ identity and flowering. Sheepgrass (*Leymus chinensis* (Trin.) Tzvel) is an important perennial forage grass and adapts well to many adverse environments. However, there are few studies on the molecular mechanisms of flower development in sheepgrass, especially studies on MADS-domain proteins.

**Results:**

In this study, we cloned 11 MADS-box genes from sheepgrass (*Leymus chinensis* (Trin.) Tzvel), and phylogenetic analysis of the 11 genes with their homologs revealed that they are divided into nine subclades. Tissue-specific expression profile analysis showed that most of these MADS-box genes were highly expressed in floral organs. *LcMADS1* and *LcMADS3* showed higher expression in the stamen than in the other tissues, and *LcMADS7* showed high expression in the stamen, glume, lemma and palea, while expression of *LcMADS2, LcMADS9* and *LcMADS11* was higher in vegetative organs than floral organs. Furthermore, yeast two-hybrid analyses showed that LcMADS2 interacted with LcMADS7 and LcMADS9. LcMADS3 interacted with LcMADS4, LcMADS7 and LcMADS10, while LcMADS1 could interact with only LcMADS7. Interestingly, the expression of *LcMADS1* and *LcMADS2* were significantly induced by cold, and *LcMADS9* was significantly up-regulated by NaCl.

**Conclusion:**

Hence, we proposed that *LcMADS1*, *LcMADS2*, *LcMADS3*, *LcMADS7* and *LcMADS9* play a pivotal role in sheepgrass sexual reproduction and may be involved in abiotic stress responses, and our findings provide useful information for further exploration of the functions of this gene family in rice, wheat and other graminaceous cereals.

**Electronic supplementary material:**

The online version of this article (10.1186/s12870-018-1259-8) contains supplementary material, which is available to authorized users.

## Background

In plants**,** MADS-box genes play important roles in many aspects of developmental processes, especially in floral induction and flower development [1]. According to their roles in flower development, MADS-box genes are classified into A, B, C, D and E classes [2, 3]. Many MADS-box genes have also been identified in rice, and MADS-box proteins determine the identity of flower organs by forming higher order complexes [4, 5]. Previous studies indicated that the A-class gene *OsMADS15* plays a role in palea development [6]. However, plants with a triple mutant of *OsMADS14*, *OsMADS15*, and *OsMADS18* have no obvious phenotype in flower development [7]. There are two B-class PI orthologous genes in rice, *OsMADS2* and *OsMADS4*, and when *OsMADS2* is silenced, transgenic plants display differences in lodicules but no changes in stamens. The *OsMADS4* gene mutant plants display no alteration in lodicule or stamen phenotypes. However, when *OsMADS2* and *OsMADS4* are silenced together, transgenic plants display palea-like structures in place of lodicules and carpel-like organs instead of stamens [8, 9]. Two B-class PI-like genes (*TaPI-1* and *TaPI-2*/*TaAGL26*) have been identified in wheat, and they are closely related to the rice PI-like genes *OsMADS4* and *OsMADS2*, respectively [10]. The C-class gene *OsMADS3* plays a role in stamen and ovule identity and is involved in lower meristem activity in early flower development and late flower development [11], while the D-class OsMADS13 is essential for *O. sativa* ovule development [12, 13]. Furthermore, expression pattern analysis demonstrates that the E-class genes specify all four whorls of floral organs and flower meristem determinacy [2, 13]. Previous studies indicated that some plant MADS-box genes are involved in abiotic stress responses. For example, *OsMADS26*, *OsMADS22* and *OsMADS55* were found to be involved in stress tolerance [14–16]. MADS-box genes have also been shown to be affected by low temperature stress in the tomato [17]. All of these findings reveal that some MADS-box genes may be involved in abiotic stress-related processes. However, until now, there have not been many reports about MADS-box gene involvement in abiotic stress responses.

Sheepgrass is a perennial forage grass with high protein content, vegetative productivity and palatability [18]. It can adapt well to many adverse environmental conditions, including drought, high salinity and alkalinity, and cold [19]. In sheepgrass, the basic unit of the inflorescence is the spikelet, and a spikelet contains two glumes and a number of flowers, which is similar to barley or wheat [20, 21]. The MADS-domain proteins are involved in many developmental processes in plants (e.g., floral organ identity and flowering). However, there are few studies on MADS-domain proteins in sheepgrass.

In this study, 11 MADS-box genes were cloned and further studied. A phylogenetic tree combining MADS proteins from sheepgrass and other species was constructed to examine their evolutionary relationships. MADS-box genes of sheepgrass were differentially expressed in vegetative and reproductive organs, and the interaction relationships of the MADS-box genes were validated by yeast two-hybrid assays. At the same time, the abiotic stress-induced expression patterns of these genes were also analyzed by qRT-PCR. Our work suggested that the MADS-box genes in sheepgrass are involved in sexual reproduction and abiotic stress and will also complement previous reports on the functions of this gene family in Triticeae.

## Methods

### Plant materials, growth conditions and stress treatments

Two-year-old sheepgrass of the variety Zhongke 1 was used for the experiments. Sheepgrass was planted under natural conditions in a field at the Beijing Botanical Garden, Chinese Academy of Sciences, Beijing, China (40° 07′ N, 116° 11′ E). The leaves, stems, roots and flower tissues including glumes, lemmas, paleas, stamens and carpels were collected at the time of flowering. All samples were immediately frozen in liquid nitrogen and stored at − 80 °C for tissue-specific expression analysis. For stress treatments, seeds were grown in a soil mixture containing 2:1 peat moss and vermiculite (*v*/v) in a greenhouse at 22–27 °C under 16-h light/8-h dark conditions. The seedlings of four-week-old sheepgrass were used for different stress treatments. For cold treatment, seedlings were placed in a chamber at 4 °C. For the ABA, drought and salt treatments, seedlings were irrigated with 100 μM ABA, 300 mM mannitol and 200 mM NaCl, respectively. Plants were sampled at the 0, 4th, 8th and 12th hour after abiotic stress treatments and immediately frozen in liquid nitrogen for further analysis.

### Cloning of the 11 MADS-box genes from sheepgrass

**Fig. 1:**
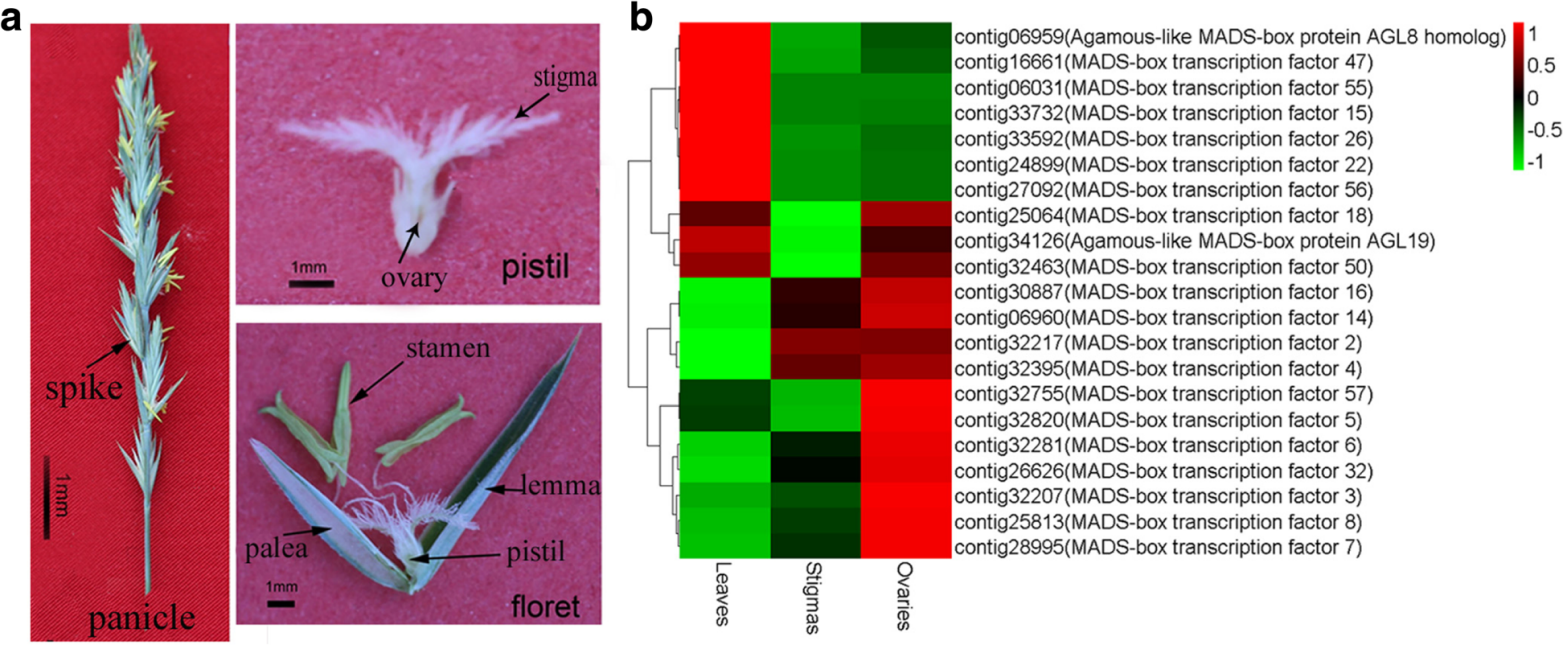
The transcript abundance profiles of MADS-box genes in sheepgrass. (**a**) The structural representation of floral organs of *Leymus chinensis*. (**b**) The expression profile of 21 putative genes

To clone the full-length sequence of the MADS-box genes, total RNA was isolated from 100 mg of frozen inflorescences (Fig. 1a) using a TRIzol kit (TaKaRa, Dalian, China) according to the manufacturer’s instructions. Gene-specific primers were designed and used to amplify the full-length coding sequences (CDS) (Additional files 1: Table S1). The amplification conditions were as follows: 95 °C for 5 min, followed by 38 cycles of 95 °C for 30 s, 56 °C for 30 s, 72 °C for 1 min, and a final extension at 72 °C for 7 min. All the PCR products were purified, ligated into a pMD18-T vector (TaKaRa, Dalian, China) and sequenced.

### Phylogenetic analysis

Eleven *L. chinensis* MADS-box predicted amino acid sequences and 35 related MADS-box genes in other plant species (five *Arabidopsis* MADS-box genes, twelve *O. sativa* MADS-box genes, four *H. vulgare* MADS-box genes, three *Aegilops tauschii subsp. tauschii* MADS-box genes, eleven *T. aestivum* MADS-box genes, one *B. distachyon* MADs-box gene) were selected to infer their evolutionary relationships using the maximum likelihood method in MEGA 6.0 [22, 23]. The other confirmed MADS-box protein sequences were obtained by a BLAST search from the National Center for Biotechnology Information (NCBI). The software DNAMAN was used to perform a multiple protein sequence alignment of 11 LcMADSs and 10 rice MADS-box factors.

### RNA extraction and cDNA synthesis

Total RNA from different tissues, including glume, lemma, palea, stamen, carpel root, stem, and leaf, was isolated using an RNAiso Plus kit (Takara, Japan), according to the manufacturer’s instructions, and treated with DNase I (Takara, Japan). The quality of RNA was determined using 1% agarose gel electrophoresis. Then, the acceptable RNA was treated with DNase I (Takara, Japan) at 37 °C for 20–30 min. The cDNA was synthesized using the PrimeScript^®^ RT Reagent Kit (TaKaRa, Dalian, China).

### Real-time RT-PCR analysis

To analyze the expression profiles of these 11 MADS-box genes in different organs and under different abiotic stress conditions, quantitative real-time polymerase chain reaction (qRT-PCR) was performed using a LightCycler 480 System (Roche, Germany). For the qRT-PCR, the cDNA was diluted 1:50 with EASY Dilution (Takara, Japan). Each reaction contained 20 μl (10 μl SYBR Premix Ex Taq II (2×), 2.0 μl cDNA, 0.8 μM of each primer and 6.4 μl ddH_2_O). The following program was performed: 95 °C for 30 s, 40 cycles of 95 °C for 5 s and 68 °C for 20 s. Three biological and three technical replicates were executed with *LcACTIN* as the internal control. The results of the qRT-PCR were analyzed with the 2^-ΔΔCt^ method, and all primers used in this study are listed in Additional files 1: Table S1.

### Yeast 2-hybrid analysis

Yeast two-hybrid (Y2H) analysis was performed to investigate the interactions among the 11 MADS-box proteins. Yeast two-hybrid analysis was performed using the GAL4 system (Clontech, Mountain View, CA, USA). The ORFs of the 11 MADS-box genes were inserted into the pGADT7 and the pGBKT7 vectors and co-transformed into *Saccharomyces cerevisiae* strain AH109 to express the fusion proteins. The positive transformants were selected on SD/−Trp-Leu medium and confirmed by PCR. The empty vectors were used as a negative control. The interactions among these 11 MADS-box proteins were assayed on SD/−Trp-Leu-His-Ade medium supplied with 3 mM 3-AT at 30 °C for 4–6 d. For the autoactivation test, the single transformants with pGBKT7 were tested by incubation on SD select medium (SD-His-Trp + 0, 1, 2, 3, 4, 5 or 10 mM 3-AT).

## Results

### Cloning and sequence analysis of 11 MADS-box genes in sheepgrass

As shown in Fig. 1a, the fertile floret of sheepgrass consists of two bract-like structures (a lemma and a palea), two lodicules, three stamens, and one pistil (stigma and ovary) from the outside to the inside. In our previous study, transcriptome sequencing techniques were used to study the self-incompatibility mechanisms of sheepgrass. Compared with mature stigmas, ovaries and leaves, we identified 1025 specifically or preferentially expressed genes of mature stigmas [24]. Based on the transcriptome data, we found that the transcript profiles of 21 putative MADS-box gene sequences were significantly different in the three tissues of sheepgrass (Fig. 1b, Additional files 2: Table S2), and among them, 7 MADS-box gene sequences were only highly expressed in ovaries, 4 were highly expressed in both stigmas and ovaries, and 3 were highly expressed in ovaries and leaves. However, 7 MADS-box gene sequences were obviously highly expressed in leaves.

Furthermore, the full-length coding sequences (CDS) of 11 MADS-box genes were cloned and named *LcMADS1, LcMADS2, LcMADS3, LcMADS4, LcMADS5, LcMADS6, LcMADS7, LcMADS8, LcMADS9, LcMADS10* and *LcMADS11*. The CDS and predicted amino acid sequences of these 11 *LcMADS* genes are listed in Additional files 3: Table S3, and all the sequences were submitted to NCBI (GenBank No. 1963149).

**Fig. 2:**
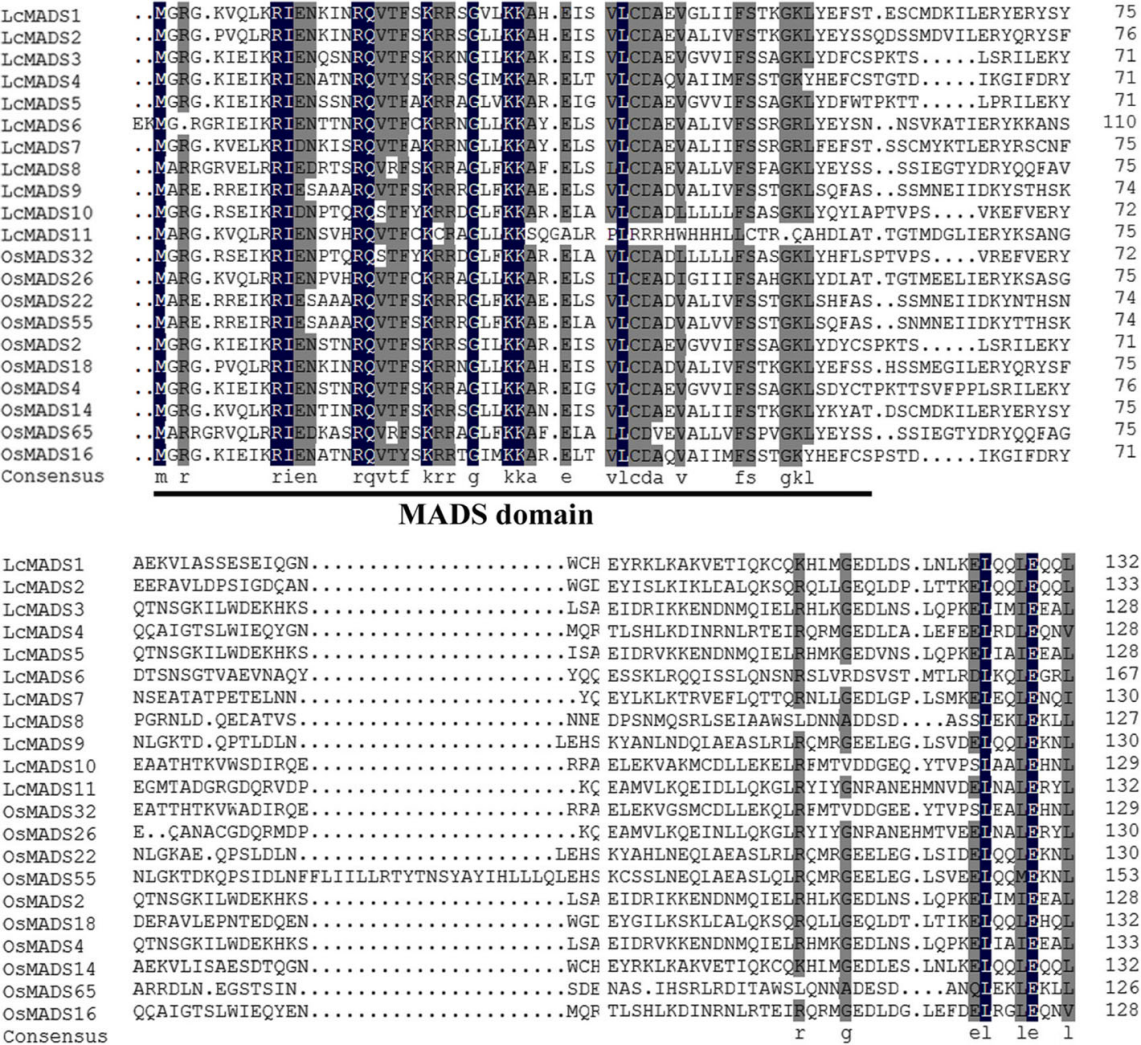
Multiple sequence alignment of sheepgrass MADS-box proteins with their corresponding proteins in *O. sativa*. The GenBank accession numbers of the genes used are shown in Additional files 4: Table S4

Multiple sequence alignment indicated that the 11 sheepgrass MADS-box genes had the typical MADS domain structure in their coding proteins, and the MADS domain that localized in the N-terminus of each protein was highly conserved (Fig. 2). In addition, these sheepgrass MADS-box genes showed high consistency with their orthologs in *Oryza sativa* (Fig. 2).

### Phylogenetic analysis

**Fig. 3:**
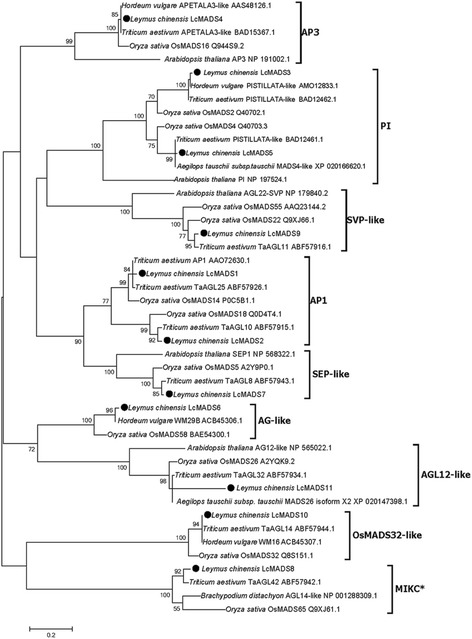
Evolutionary relationships of MADS-domain proteins from sheepgrass and other species. The analysis involved 46 amino acid sequences

To determine the evolutionary relationship between the 11 sheepgrass MADS-box genes and those of other species, a phylogenetic tree was created according to the maximum likelihood method. Our results showed that these 11 MADS-box proteins in sheepgrass belonged to nine clades and shared high similarity with the *T. aestivum* or *H. vulgare* MADS-box sequences. The phylogenetic analysis clearly confirmed that *LcMADS1* and *LcMADS2* were closely related to *OsMADS14* and *OsMADS18,* respectively, and each of these genes belonged to the AP1-like subfamily (A-class), which is involved in specifying sepals and petals in *Arabidopsis* [25]. *LcMADS3, LcMADS5* and *LcMADS4,* together with their homologs *OsMADS2*, *OsMADS*4 and *OsMADS16,* were assigned into the PI and AP3 clades (B-class), indicating that these three sheepgrass MADS-box genes function in the development of lodicules and stamens. *LcMADS7*, *LcMADS8*, *LcMADS9* were divided into the SEP-like, MIKC^*^-type, and SVP-like clades, respectively (Fig. 3).

### Tissue-specific expression analysis of 11 sheepgrass MADS-box genes

**Fig. 4:**
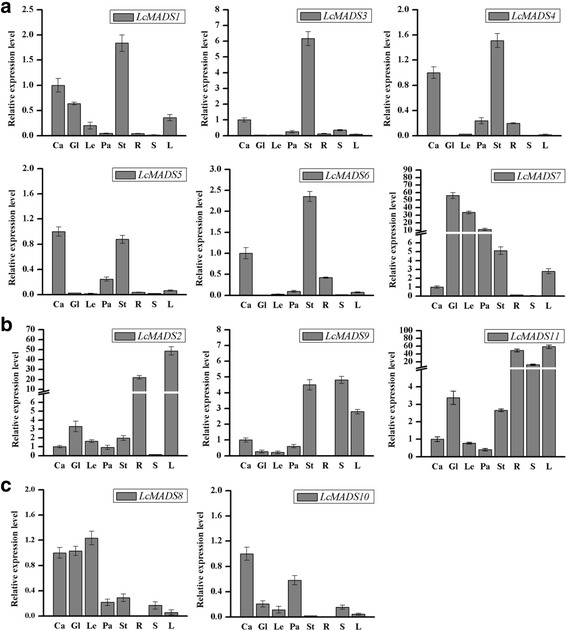
Expression patterns of sheepgrass MADS-box genes in vegetative and reproductive organs based on qRT-PCR analyses. (**a**) Six *LcMADS* genes had higher expression in stamens. (**b**) Three L*cMADS* genes had higher expression in vegetative organs. (**c**) Two L*cMADS* genes had higher expression in carpels. Sources of the samples are as follows: root (R), stem (S), leaf (L), carpel (Ca), glume (Gl), lemma (Le), palea (Pa) and stamen (St)

Based on the differential expression results of these MADS-box genes in the heat map analysis, we found that the expression of MADS-box genes was significantly different in three tissues of sheepgrass (Fig. 1b). To further investigate the expression patterns of the 11 MADS-box genes in different tissues and organs, we harvested the root, stem, leaf, and floral organs, which included glumes, lemmas, paleae, stamens and carpels for qRT-PCR. The results showed that most of these MADS-box genes in sheepgrass were highly expressed in the floral organ (Fig. 4a). PI-like genes *LcMADS3* and *LcMADS5* were highly expressed in stamens and carpels, but weak expression was detected in vegetative organs. The SEP-like gene *LcMADS7* showed high expression in all floral organs, such as stamens, glumes, lemmas and paleas. Interestingly, the expression of the *LcMADS2, LcMADS9* and *LcMADS11* genes was higher in vegetative organs (stem, leaf and root) than in floral organs (Fig. 4b), while the transcript abundance of *LcMADS8* and *LcMADS10* was higher in carpels than other tissues or organs (Fig. 4c**)**.

### MADS-box genes from sheepgrass involved in abiotic stress

Previous studies showed that some MADS-box genes are involved in stress tolerance [2], and our study investigated the responses of 11 MADS-box genes to abiotic stresses in sheepgrass. In this study, seedlings were exposed to 100 μM ABA, 300 mM mannitol, 200 mM NaCl, or a low-temperature treatment (4 °C).

**Fig. 5:**
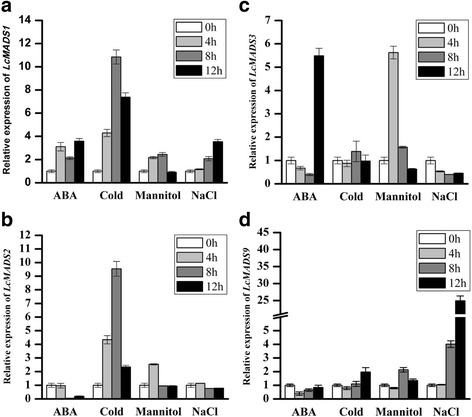
Differential expression levels of four MADS-box genes in response to cold, salt, drought and ABA stress. Relative expression levels of (**a**) *LcMADS1*, (**b**) *LcMADS2*, (**c**) *LcMADS3*, and (**d**) *LcMADS9* are shown. The transcript levels of the *LcMADS* genes at 0 h were used as controls

**Fig. 6:**
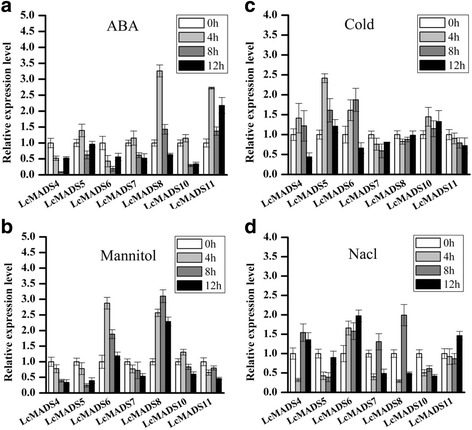
Differential expression of seven MADS-box genes in response to ABA (**a**) drought (**b**), cold (**c**) and salt (**d**) stress. The transcript levels of *LcMADS* genes at 0 h were used as controls

As a whole, *LcMADS1*, *LcMADS2*, *LcMADS3*, and *LcMADS9* were significantly induced by abiotic stresses (Fig. 5). *LcMADS1* and *LcMADS2* were strongly induced by cold (Fig. 5a, b). *LcMADS3* was up-regulated when treated with ABA and mannitol, and its expression levels were higher at 12 h in the ABA treatment and at 4 h in the mannitol treatment (Fig. 5c). Furthermore, *LcMADS9* was significantly induced by NaCl and its expression level reached its peak at 12 h (Fig. 5d). We also analyzed the stress-induced expression profile of the other *LcMADS* genes in sheepgrass, and our results indicated that *LcMADS8* and *LcMADS11* were up-regulated when treated with ABA, while *LcMADS4* was down-regulated by ABA and mannitol treatment (Fig. 6a, b). However, the differences in the responses to different stresses were not significant for *LcMADS5*, *LcMADS7*, *LcMADS10* or *LcMADS11* (Fig. 6). Thus, we suggested that some MADS-box genes in sheepgrass may also be involved in the abiotic stress response as an escape strategy.

### Interaction analysis of 11 sheepgrass MADS-box proteins revealed by the yeast two-hybrid assay

**Fig. 7:**
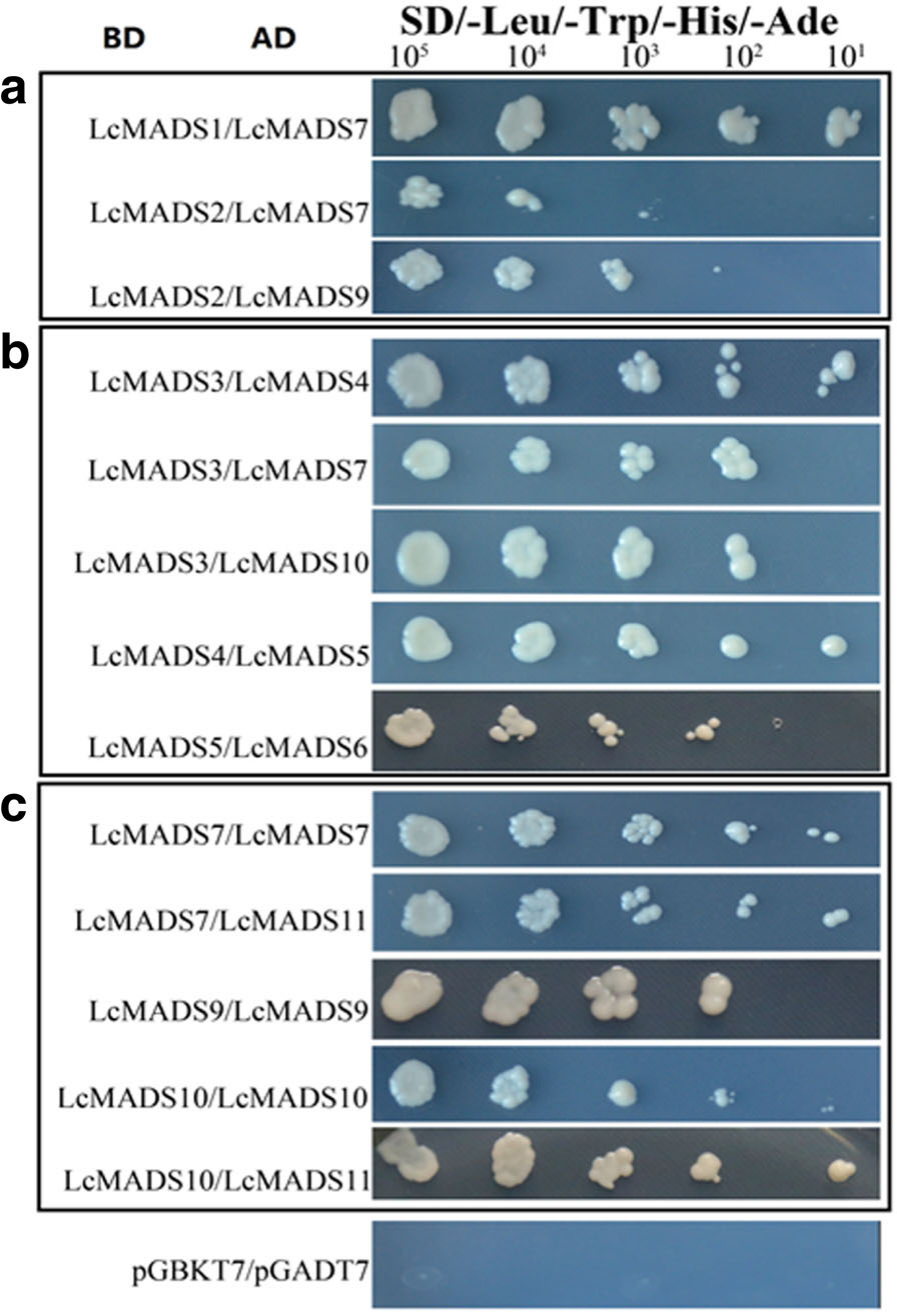
Yeast two-hybrid assays of 11 MADS-box proteins of sheepgrass. Protein-protein interactions of (**a**) two A-class MADS-box proteins, (**b**) three B-class MADS-box proteins, and (**c**) three other classes of MADS-box proteins. Serial dilutions (10^5^–10^1^) of AH109 cells containing different plasmid combinations were grown on the selective medium plates SD/−Trp-Leu-His-Ade/3 mM 3-AT

Protein interactions are essential not only for the normal roles that proteins play but also for expanding the functional diversities of proteins [26]. The yeast two-hybrid assay is an effective method to discover interaction relationships in vitro and to understand molecular networks. We performed the yeast two-hybrid assays to investigate the protein-protein interaction relationships among the 11 MADS-box genes (two A-class genes, three B-class genes, one C-class gene, one SEP gene, one *AGL12*-like gene, one *OsMADS32*-like gene, one *SVP*-like gene and one MIKC*-type gene) in sheepgrass. No self-activation was observed for any of the single constructs with pGBKT7 on SD selective medium (SD-His-Trp + 3 mM 3-AT). Of all the combinations of the 11 MADS-box proteins, only 13 combinations showed positive results on SD/−Trp-Leu-His-Ade medium (Fig. 7). The results showed that the A-class proteins LcMAD1 and LcMAD2 could both interact with the E-class protein LcMAD7, and LcMAD2 could also interact with the SVP-like LcMAD9 protein (Fig. 7a). The interaction of proteins of three B-class proteins, LcMADS3, LcMADS4, and LcMADS5, are shown in Fig. 7b. The results indicated that LcMADS3 interacted with LcMADS4 and could also interact with LcMADS10 and LcMADS7. LcMADS4 could interact with another B-class protein, LcMADS5. However, LcMADS5 interacted with the C-class protein LcMADS6 (Fig. 7b). In addition, our results showed that LcMADS7 could interact with four LcMADSs, but the interaction between LcMADS2 and LcMADS7 was very weak. LcMADS10, LcMADS9 and LcMADS7 could form homodimers, and the MIKC*-type gene LcMADS8 could not interact with any other LcMADSs (Fig. 7c).

## Discussion

In the plant ABC (DE) model, MADS-box genes are critical transcription factors that are involved in floral organ identity specification [3, 27, 28]. A-, B-, C-, D-, and E-class genes have been confirmed in grass species, and there are four A-class genes, three B-class genes, two C-class genes, two D-class genes, and seven E-class genes (homologs of SEP and AGL6 genes in eudicots) in the *O. sativa* genome [11, 29–31]. Sheepgrass is a member of the tribe Triticeae and is one of the important perennial forage grasses with high quality and stress resistance in China [19, 32, 33]. In our previous studies, a large set of 1025 genes specifically expressed in the stigma was identified [24], and 21 putative MADS-box gene sequences were found in sheepgrass (Fig. 1b, Additional files 2: Table S2). Based on the transcriptome sequencing data, we identified 11 sheepgrass MADS-box genes (Fig. 2, Additional files 3: Table S3). These genes encoding proteins were divided into nine clades based on their evolutionary relationships, and all of them have close orthologs in *H. vulgare*, *T. aestivum* and *O. sativa*, indicating the large extent of conservation among the sheepgrass and other grass MADS-box gene families. Furthermore, two genes belong to the A class, and three genes belong to the B class (Fig. 3). This finding will provide valuable information for further studies of the functions of different MADS-box genes classes in sheepgrass.

Previous studies suggested that the A-class (AP1 and AP2) MADS-box genes are specific to the outermost sepals and that carpels are controlled by the C (AG)-class genes, while the combination of A-, B- (AP3 and P1) and E-class genes controls petal identity [34–36]. A large number of SEP-like genes have also been identified in monocots [37, 38]. In maize, there are at least eight SEP-like genes with distinguishable expression patterns that most likely reflect diverse functions [39]. In this study, the tissue expression patterns of 11 MADS-box genes revealed that most of these MADS-box genes in sheepgrass were highly expressed in floral organs, similar to the expression profiles of their homologous genes in *T. aestivum* and *O. sativa*. The tissue-specific expression analysis indicated that the *LcMADS1* gene was expressed higher in stamens, carpels, and glumes (Fig. 4a), while the functions of their homologs in rice *OsMADS14* were not only involved in specifying meristem identity but also palea and lodicule identities [5]. One AP1-like gene, *LcMADS2,* was expressed in reproductive organs similar to the expression of its homolog in *T. aestivum* and was also expressed in the leaf and root (Fig. 4b), consistent with the expression of its homolog *OsMADS18* [29]. Consistent with the expression patterns of PI in *Arabidopsis*, homologues in sheepgrass *LcMADS3* and *O. sativa OsMADS2* were also clearly expressed in the stamen and the carpal (Fig. 4a) [9, 40]. The *LcMADS9* gene had high homology with rice *OsMADS22* and *OsMADS55*, and overexpression of *OsMADS22* and *OsMADS55* led to abnormal floral morphologies including leaf-like sepals [41]. However, *OsMADS22* is expressed in non-vegetative tissues, and its ectopic expression induces spikelet meristem indeterminacy [42], while *LcMADS9* was not only expressed higher in flowers but also expressed relatively highly in vegetative organs, such as the stem and leaf (Fig. 4b). In addition, the expression pattern of the SEP-like gene *LcMADS7* was strongly expressed in the glume, lemma, palea and stamen (Fig. 4c).

In previous reports, some plant MADS transcription factors acted as crucial regulators in response to abiotic stresses [43]. For example, MADS-box genes have been shown to be affected by low temperature, photoperiod, and plant hormones such as cytokinins, gibberellins and ethylene [17, 44–46]. In this study, we found that the A-class *LcMADS1* and *LcMADS2* genes were both significantly up-regulated under cold stress (Fig. 5a, b), while the B-class *LcMADS3* gene was found to exhibit high expression in response to mannitol (4 h) and ABA (12 h) (Fig. 5c). These results suggested the two classes of genes function in different stress responses. We found that *LcMADS9* was induced by salt (Fig. 5d), while its homolog *OsMADS22* exhibited a different expression pattern; it was up-regulated by more than two-fold in response to cold and dehydration treatments [2]. In *O. sativa*, *OsMADS26*, an AGL12-class gene, has also been reported to be involved in drought tolerance [15], and its ortholog *LcMADS11* was up-regulated by ABA (Fig. 6a). Taken together, our results revealed novel roles of *LcMADS* genes in response to abiotic stresses and may provide useful clues for future research on grass *MADS* family gene responses to abiotic stress signaling processes.

**Fig. 8:**
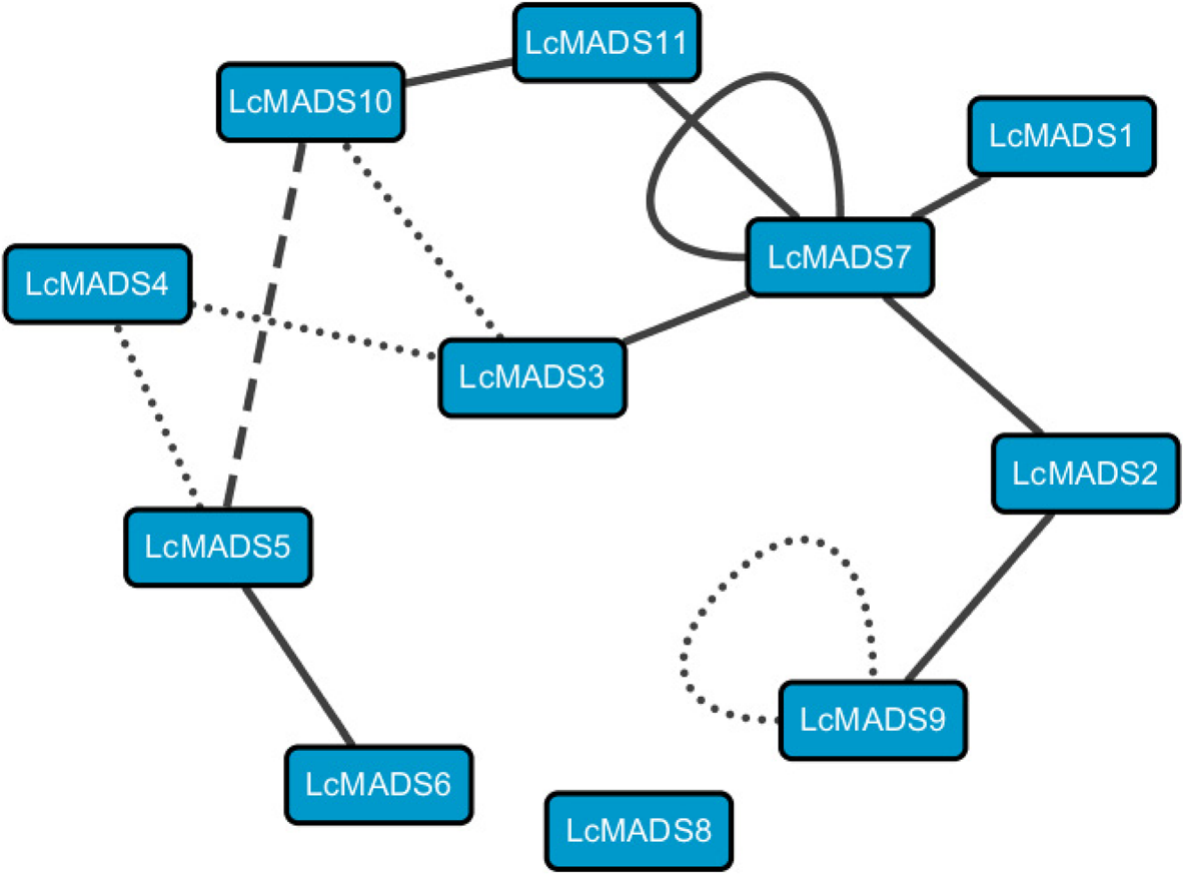
Interaction maps of putative orthologous MADS-domain proteins from sheepgrass and rice. The putative orthologs were the closest homologs derived from phylogenetic analysis. Each node represents putative orthologous proteins from two species. The nodes were named using sheepgrass MADS-domain proteins. Thick solid line, dashed dot line, and dotted line represent putative orthologous proteins were interacted in sheepgrass, rice, and both species, respectively

Interactions between MADS-box proteins are central to the ABCDE model of flower formation and development [26, 47]. In *Arabidopsis*, SEP proteins can directly interact with ABC MADS-domain proteins and act as bridges for higher-order complexes [4, 48]. Here, we used yeast two-hybrid systems to investigate the protein-protein interactions among 11 sheepgrass MADS box proteins (Fig. 7), and a composite figure was used to understand their molecular networks and provide a framework for the interaction capacity for these MADS-box proteins in sheepgrass and rice (Fig. 8). The direct interaction of B-class proteins with the SEP subfamily proteins has been demonstrated in the chrysanthemum and the tomato [47, 49, 50], and our study reconfirmed that B-class proteins could interact with SEP proteins. Interestingly, orthologues of LcMADS3 were able to interact with LcMADS4 and LcMADS10 in both sheepgrass and rice (Fig. 8). Phylogenetic analysis showed that *LcMADS3* and *OsMADS2* belonged to PI-class genes (Fig. 3). OsMADS2 was able to interact with the AP3 protein OsMADS16 (which had a close relationship with LcMADS4) and OsMADS32 (homologous to LcMADS10) [51, 52], and the LcMADS3 protein displayed the same interaction patterns in sheepgrass (Fig. 8, dotted line). Furthermore, the SEP-like protein LcMADS7 had an extensive interaction network that included PI-like (LcMADS3), AP1-like (LcMADS1 and LcMADS2), and AGL12-like proteins (LcMADS11), and it also formed a homodimer (LcMADS7). However, the SEP-like OsMADS5 protein cannot homodimerize or heterodimerize with other SEP proteins [53]. Two proteins, LcMADS1 and LcMADS2, belonged to the AP1 clade, and both of these proteins interacted with LcMADS7, while LcMADS2 could also interact with LcMADS9 (Fig. 7). LcMADS1 and LcMADS2 were recently identified and exhibited homology to OsMADS14 and OsMADS18, respectively (Fig. 8). Whether their paralogs in *O. sativa* has the same protein interaction patterns requires further verification. Hence, these SEP-like proteins of sheepgrass and *O. sativa* had different interaction partners, indicating that SEP-like proteins might have potentially novel functions in sheepgrass.

## Conclusion

We first cloned 11 MADS-box genes in sheepgrass, which play important roles in flower development, and taken together, our results showed the expression patterns of *LcMADs* genes in various tissues and under different abiotic stress conditions. Our results indicated that the MADS-box genes *LcMADS1* and *LcMADS3* were highly expressed in sheepgrass stamens. The expression levels of *LcMADS2* and *LcMADS9* were high in vegetative tissues. Meanwhile, *LcMADS1*, *LcMADS2*, *LcMADS3* and *LcMADS9* were significantly induced by abiotic stresses. In addition, we first demonstrated the interaction relationship between 11 sheepgrass MADS-box proteins, and our results indicated that LcMADS2 interacted with LcMADS7 and LcMADS9. LcMADS3 interacted with LcMADS4, LcMADS7 and LcMADS10, while LcMADS1 could interact with only LcMADS7. LcMADS7 could interact with four LcMADSs. Hence, we proposed that *LcMADS1*, *LcMADS2*, *LcMADS3*, *LcMADS7* and *LcMADS9* play a pivotal role in sheepgrass sexual reproduction and may be involved in abiotic stress responses. Our findings provide useful information for further exploration of the functions of this gene family in rice, wheat and other graminaceous cereals.

## Additional files

Additional file 1: **Table S1.** All primers used in this study. (XLSX 11 kb)

Additional file 2: **Table S2.** The expression information of 21 putative MADS-box gene in stigma, leaf, ovary of sheepgrass based on the transcriptome data. (XLSX 11 kb)

Additional file 3: **Table S3.** The sequences of 11 Sheepgrass MADS-box genes. (XLSX 15 kb)

Additional file 4: **Table S4.** The GeneBank accession numbers of genes used in multiple sequence alignment. (XLSX 10 kb)

## Abbreviations

ABA: Abscisic acid
BLAST: Basic local alignment search tool
cDNA: Complementary DNA
CDS: Coding sequences
LcMADSs: *Leymus chinensis* MADS-box genes
NaCl: Sodium chloride
NCBI: The national center for biotechnology information
qRT-PCR: Quantitative real-time polymerase chain reaction
SEP: SEPALLATA
